# Haematopoetic stem cell transplantation at apollo group hospitals

**DOI:** 10.1186/1755-8166-7-S1-I60

**Published:** 2014-01-21

**Authors:** Chirag Shah

**Affiliations:** 1 Apollo Hospitals International Limited, Gandhinagar, Gujarat, India

## 

Hematopoietic stem cell transplantation (HSCT) is the transplantation of multipotent hematopoietic stem cells, usually derived from bone marrow, peripheral blood, or umbilical cord blood. It is a medical procedure in the fields of hematology and oncology, most often performed for patients with certain cancers of the blood or bone marrow, such as multiple myeloma or leukemia. HSCT can be divided in two types, autologous and allogenic transplant based on the source of stem cells. Autologus is when stem cells are collected from patient’s own body. Allogeneic is when stem cells are collected from someone else.

Apollo group hospitals perform largest number of HSCT in private sector. HSCT requires multidisciplinary effort with team of many experts including haematologist/oncologist, intensive care specialist, trained nursing staff, experts for venous access, infectious disease specialist, transfusion medicine expert, counsellors and many other experts.

Over 700 patients have been treated by HSCT. The majority of these patients have haematological malignancies, consisting of acute and chronic leukaemias, lymphomas, plasma cell myeloma and other haematological neoplasms. Patients with other benign blood diseases including thalassemia, sickle cell disease, aplastic anemia, Fanconi anemia, and others have also been treated. The major facility for these patients is a specially designed haematology ward with HEPA-filtered air.

The hemato-oncology department manages both adult and paediatric haematopoietic stem cell transplantation cases, where more than a hundred allogeneic and autologous transplantations are now performed every year. The full range of allogeneic transplantation is performed, with haematopoietic stem cells coming from HLA-identical siblings, matched unrelated donors, umbilical cord blood and haploidentical donors.

